# Detection of Enterotoxigenic Potential and Determination of Clonal Profile in *Staphylococcus aureus* and Coagulase-Negative Staphylococci Isolated from Bovine Subclinical Mastitis in Different Brazilian States

**DOI:** 10.3390/toxins8040104

**Published:** 2016-04-15

**Authors:** Priscila Luiza Mello, Danilo Flávio Moraes Riboli, Luiza Pinheiro, Lisiane de Almeida Martins, Maria Aparecida Vasconcelos Paiva Brito, Maria de Lourdes Ribeiro de Souza da Cunha

**Affiliations:** 1 Department of Microbiology and Immunology, Institute of Biosciences of Botucatu, UNESP-Univ Estadual Paulista, Botucatu 18618-970, Brazil; priscila_mello@ibb.unesp.br (P.L.M.); danilo.riboli@ibb.unesp.br (D.F.M.R.); luizapinheiro@ibb.unesp.br (L.P.); 2 Department of Anatomic Pathology, Instituto Lauro de Souza Lima, Bauru 17034-971, Brazil; 3 Universidade Paranaense-UNIPAR, Umuarama 87502-210, Brazil; lisiane.almeida.martins@gmail.com; 4 Embrapa Dairy Cattle, Juiz de Fora 36038-330, Brazil; maria.brito@embrapa.br

**Keywords:** staphylococcal enterotoxin, bovine mammary gland, Brazilian States, mastitis, pulsed-field gel electrophoresis, clonal profile

## Abstract

Epidemiological studies have identified *Staphylococcus aureus* as the most common agent involved in food poisoning. However, current research highlights the importance of toxigenic coagulase-negative staphylococci (CoNS) isolated from food. The aim of this study was to characterize *Staphylococcus* spp. isolated from cows with bovine subclinical mastitis regarding the presence of genes responsible for the production of staphylococcal enterotoxins and of the *tst-*1 gene encoding toxic shock syndrome toxin 1, and to determine the clonal profile of the isolates carrying any of the genes studied. A total of 181 strains isolated in different Brazilian states, including the South, Southeast, and Northeast regions, were analyzed. The *sea* gene was the most frequent, which was detected in 18.2% of the isolates, followed by *seb* in 7.7%, *sec* in 14.9%, *sed* in 0.5%, *see* in 8.2%, *seg* in 1.6%, *seh* in 25.4%, *sei* in 6.6%, and *ser* in 1.6%. The *sej*, *ses*, *set*, and *tst-*1 genes were not detected in any of the isolates. The typing of the isolates by pulsed-field gel electrophoresis revealed important *S. aureus* and *S. epidermidis* clusters in different areas and the presence of enterotoxin genes in lineages isolated from animals that belong to herds located geographically close to each other.

## 1. Introduction

Several microorganisms are involved in mammary gland infections in production animals. National and international epidemiological studies have demonstrated the presence of the genus *Staphylococcus* in approximately 50% of bovine mastitis cases, highlighting the role of this group as the main causative agent of this infection [1]. Although *Staphylococcus aureus* is the most common agent involved in subclinical mastitis, coagulase-negative staphylococci (CoNS) have gained importance as causative agents of intramammary infections. In a study conducted in Brazil, Rall *et al.* [2] found a prevalence of these microorganisms of 27.4%, while 28.6% of the isolates were *S. aureus* in a study conducted in Turkey [3].

Studies [4,5] have emphasized the importance of toxigenic CoNS isolated from food. Therefore, although *S. aureus* is the most common agent involved in food poisoning, there is current concern in the scientific community regarding CoNS, which have been recognized as opportunistic pathogens in human and animal infections, allied to risks of toxigenic lineages in cases of food poisoning in humans [5].

The staphylococcal enterotoxins (SE) type A (*sea*), B (*seb*), C (*sec*), D (*sed*), and E (*see*) are the classical staphylococcal toxins. These toxins have emetic activity and are usually associated with outbreaks of food poisoning [6]. In addition, recently described enterotoxins (*ses* and *set*), enterotoxin-like (SE-like) toxins that do exert emetic activity, and toxic shock syndrome toxin 1 (*tst*-1) are known [6,7]. The staphylococcal enterotoxin R (*ser*) was first described as an enterotoxin-like toxin by Omoe *et al*. [6], but experimental studies later proved its emetic activity [8].

The objective of this study was to characterize the enterotoxigenic potential of *Staphylococcus* spp. isolated from cattle with subclinical mastitis in six Brazilian states: Paraná (PR), Santa Catarina (SC), Rio Grande do Sul (RS), São Paulo (SP), Minas Gerais (MG), and Pernambuco (PE). Additionally, we determined the clonal profile and geographic distribution of the *S. aureus* and *S. epidermidis* strains that carried any of these genes.

## 2. Results

### 2.1. Identification of the Strains

Among the 181 strains studied, 82 (45.3%) were identified as *S. aureus* and 99 (54.7%) as CoNS, including 27 (14.9%) *S. chromogenes*, 26 (14.4%) *S. epidermidis*, 17 (9.4%) *S. saprophyticus*, 6 (3.3%) *S. warneri*, 6 (3.3%) *S. simulans*, 6 (3.3%) *S. haemolyticus*, 5 (2.8%) *S. hyicus*, 4 (2.2%) *S. hominis*, and 2 (1.1%) *S. xylosus.*

### 2.2. Detection of Enterotoxin and tst-1 Genes

**Figure 1:**
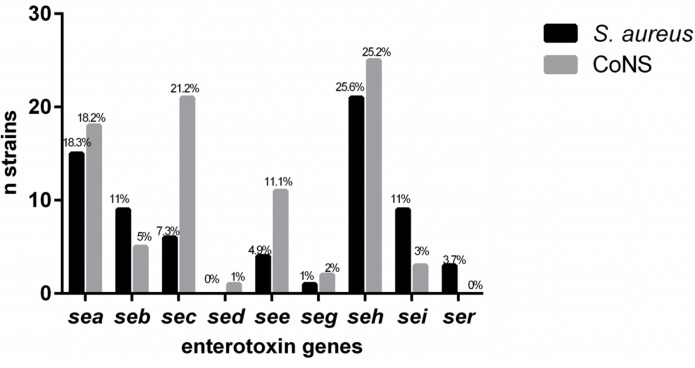
Detection of staphylococcal enterotoxins in *Staphylococcus aureus* and Coagulase-negative Staphylococci (CoNS) isolated from cows with bovine subclinical mastitis.

Seventy-six (41.9%) of the 181 strains were positive for at least one of the genes studied. The *sea* gene was detected in 33 (18.2%) of the isolates, *seb* in 14 (7.7%), *sec* in 27 (14.9%), *sed* in 1 (0.5%), *see* in 15 (8.2%), *seg* in 3 (1.6%), *seh* in 46 (25.4%), *sei* in 12 (6.6%), and *ser* in 3 (1.6%). The *sej*, *ses*, *set*, and *tst*-1 genes were not detected in any of the strains studied (Table 1). Figure 1 shows the comparison of toxin gene detection in the *Staphylococcus aureus* and CoNS isolates.

### 2.3. Distribution of Staphylococcal Enterotoxin Genes According to the Region Studied

**Figure 2:**
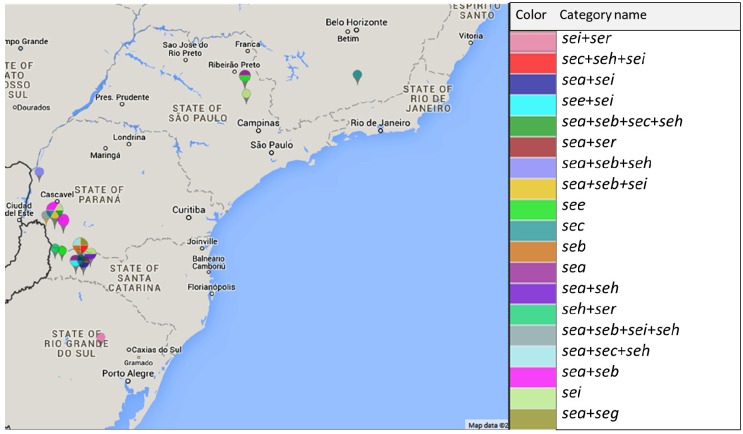
Geographic distribution of enterotoxin gene-positive *Staphylococcus* spp. strains isolated from cows with bovine subclinical mastitis in different Brazilian states.

Figure 2 shows the geographic distribution of enterotoxin gene-positive *Staphylococcus* spp. isolates. The *sea* and *seb* genes were found to coexist with the other genes studied and were detected in *Staphylococcus* spp. isolates from all Brazilian states, except for the state of Minas Gerais where only isolates carrying the *sec* gene were detected.

The *sei* and *ser* genes were only detected in *Staphylococcus* spp. from the southern states (PR, SC, and RS).

### 2.4. Determination of Clonal Profile

Molecular typing by Pulsed-field gel electrophoresis (PFGE) was performed only on the *S. aureus* and *S. epidermidis* isolates that in which any enterotoxin genes had been detected. Therefore, among the 181 strains included in the study, 27 *S. aureus* isolates and 15 *S. epidermidis* isolates were analyzed, corresponding to the species with the highest prevalence of strains carrying enterotoxin genes.

**Figure 3:**
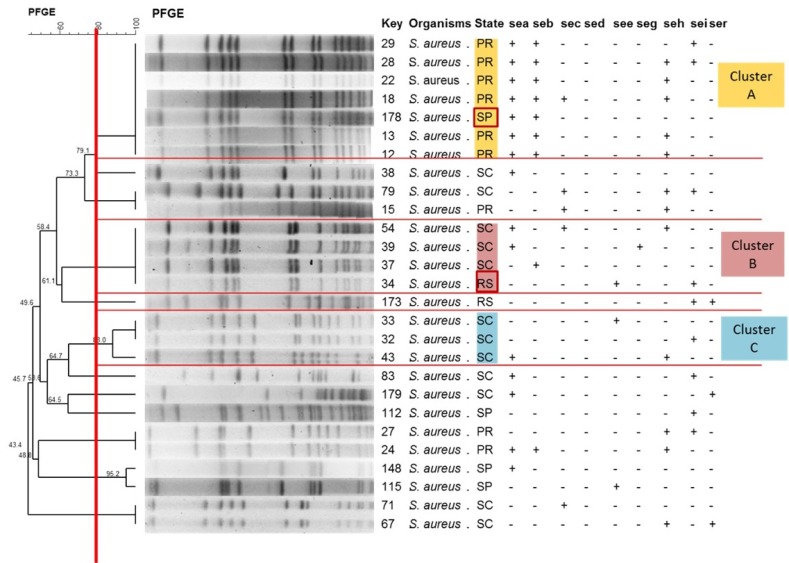
Determination of the clonal profile of *Staphylococcus aureus* strains carrying staphylococcal enterotoxin genes. The highlighted strain is interesting because of its similarity to isolates with origin in different states of Brazil (cluster A). The red lines highlight the division of the clusters and the red boxes highlight samples from different states within the cluster. Dendrogram generated by Dice/Unweighted pair group method using arithmetic averages (UPGMA) analysis (BioNumerics, Applied Maths). PR: Paraná; SP: São Paulo; SC: Santa Catarina; RS: Rio Grande do Sul.

Analysis permitted the identification of three *S. aureus* clusters that simultaneously included ≥3 isolates with similarity ≥80% (Figure 3, Table 2). Similarity was 100% in all groups. Cluster A, consisting of seven strains, contained a larger number of isolates with enterotoxigenic potential. This cluster comprised six isolates from Paraná and one isolate from São Paulo. The other clusters (B and C) also contained isolates from the South region, but from the state of Santa Catarina (Table 2). Cluster B comprised isolates originating from different states (SC and RS).

**Figure 4:**
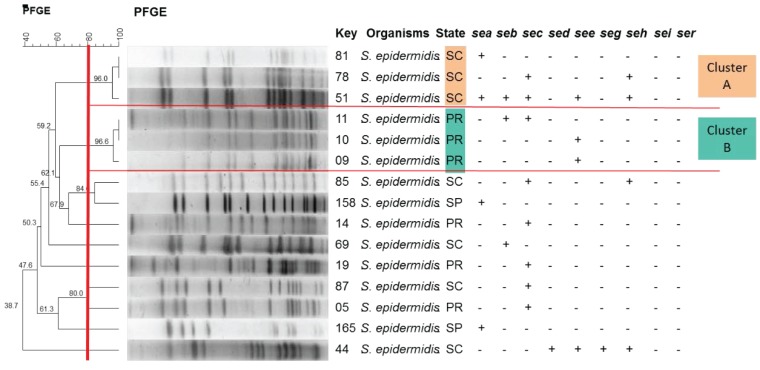
Determination of the clonal profile of *Staphylococcus epidermidis* strains carrying staphylococcal enterotoxin genes. Dendrogram generated by Dice/UPGMA analysis (BioNumerics, Applied Maths). The red lines highlight the division of the clusters.

When the profile of *S. epidermidis* was analyzed, two clusters (A and B) were found. Each cluster contained three isolates from the South region (PR and SC, respectively) (Figure 4).

## 3. Discussion

The *sea* gene, which exhibited the highest prevalence in this study (18.2%) and was detected in *S. aureus* and CoNS strains, is carried by a prophage [9] and can be easily disseminated among *Staphylococcus* spp. strains. Its product, enterotoxin A, is frequently associated with food poisoning since it is toxic at low concentrations [10,11]. Enterotoxin A is produced at the beginning of the exponential phase and its expression is not regulated by the accessory gene regulator (*agr*), different from enterotoxins B, C, and D, which depend on the *agr* system for maximum expression [11,12]. The *sec* gene is located on pathogenicity islands and can be divided into three subtypes (*sec*1, *sec*2, and *sec*3) based on antigenic differences and on the animal host with which it is associated. Some studies suggest that the heterogeneity of enterotoxin C is related to selection for modified *sec* sequences that facilitate the survival of *S. aureus* in their respective hosts [11,13]. In the present study, *sec* was the second most common classical enterotoxin after *sea*.

The *sed* gene was detected in only 0.5% of the strains studied, in an *S. epidermidis* isolate. This gene was not detected by Calsolari *et al*. [3] in toxigenic CoNS isolates, with only one *S. aureus* strain being positive. The *sed* gene is located on plasmid pIB485 [14] and enterotoxin D is the second most common toxin associated with food poisoning [11]. A small amount of this enterotoxin is able to cause illness, mainly in children and the elderly [15,16]. Nonetheless, the near absence of *sed* in the strains studied here suggests that it is scarcely related with *Staphylococcus* spp. isolates from mastitis cases in these regions of Brazil and consequently with possible events of food poisoning.

The data of a study on isolates from dairy products responsible for food poisoning in the state of Minas Gerais [16] are consistent with studies demonstrating that *sea* and *seb* are the most prevalent among the toxins identified [5]. The same study [16] showed the production of staphylococcal enterotoxins by CoNS using immunological assays.

The *see* gene was also detected in a small percentage (8.2%) and was not found in the study of Srinivasan *et al*. [17] who investigated *S. aureus* strains isolated from milk of cows with mastitis in the region of Tennessee, USA. None of the 78 isolates was positive for that gene. The *see* gene is carried by a prophage and studies have shown that the *see*, *sed*, and *sea* genes are closely related, with 81% sequence homology between *see* and *sea* [11,18].

Although the *sei* gene was only detected in a small percentage (6.6%), it was present in CoNS strains, which is an important fact rarely described in the literature. In contrast, in the study of Srinivasan *et al*. [17], 47 (60.3%) of the 78 *S. aureus* isolates studied carried the gene.

The *ser* gene was detected in three *S. aureus* isolates, all from the South regions. This gene was found to coexist with some other enterotoxins since two strains from SC carried the combination *ser* + *sea* or *ser* + *sec*, while in the third isolate from RS, *ser* was associated with the enterotoxin I gene (*sei*). These data highlight the importance of knowledge on movable genetic elements that can transfer these genes among *Staphylococcus* spp. The same has been described by Lawrynowicz-Paciorek *et al*. [19] who also evaluated the frequency of coexisting genes.

Regarding the clonal profile, several epidemiological studies on *S. aureus* in cattle have demonstrated the involvement of a large number of molecular profiles in the etiology of mastitis in the world. However, certain profiles tend to predominate in different geographic regions [19,20]. In the present study, a large number of *S. aureus* strains grouped with ≥80% similarity were observed, even when the region studied included two different states, such as clusters A and B. In both cases, the source of one isolate differed from that of the others. This similarity among isolates obtained from animals that belong to herds from different states can be explained by the trade of animals between farms.

According to Buzolla *et al*. [21], strains with identical genotypes may possess characteristics that convey advantages for their survival in the environment, to colonize the udder, and/or to cause diseases. This fact was proven in the present study of enterotoxin genes, which demonstrated the presence of clusters with the same profile in different locations. Mechanical milking machines are important sources of transmission of staphylococci in dairy herds since they can be contaminated with microorganisms derived from the animal’s skin and milk, or even from the hands of the operators [22].

Taken together, the results of clonal profile analysis showed the existence of important genetic similarity among the isolates, particularly among *S. aureus* isolated from intramammary infections of herds from different Brazilian states. Furthermore, PFGE was found to be an excellent technique to detect these clones, permitting the establishment of emergency control measures to prevent subsequent dissemination of virulent strains.

## 4. Experimental Section

### 4.1. Origin and Identification of the Strains

The 181 *Staphylococcus* spp. strains isolated from cows with bovine subclinical mastitis were provided by Embrapa Dairy Cattle. The strains were isolated in six Brazilian states: Paraná (PR), Santa Catarina (SC), and Rio Grande do Sul (RS); São Paulo (SP) and Minas Gerais (MG); and Pernambuco (PE), corresponding to the South, Southeast, and Northeast regions of Brazil, respectively.

The phenotypic and genotypic identification of these strains was performed for confirmation of the genus and correct identification of the species. The genus *Staphylococcus* was identified as described by Koneman *et al*. [23] using coagulase and sugar (trehalose, maltose, and mannitol) fermentation tests. Strains belonging to the CoNS group were submitted to biochemical tests as proposed by Cunha *et al*. [24] for phenotypic identification of the species. Total DNA was extracted using the illustra^®^ kit (GE Healthcare, Little Chalfont, Buckinghamshire, UK). Genotypic identification of CoNS was performed using primers targeting conserved sequences adjacent to the 16S and 23S genes by the internal transcribed spacer-polymerase chain reaction (ITS-PCR) described by Barry *et al*. [25] and Couto *et al*. [26], using primers G1 and L1. PCR using the Staur-4 and Staur-6 primers developed by Straub *et al*. [27] was used for *S. aureus*. The *S. aureus* ATCC 33591 reference strain was used as the positive control. The amplification efficiency was monitored by 2% agarose gel electrophoresis stained with Saber Safe DNA Gel Strain^®^ (São Paulo, SP, Brazil) viewed under a UV transilluminator.

The reference strains used for ITS were: *S. chromogenes* (ATCC 43764), *S. epidermidis* (ATCC 12228), *S. saprophyticus* (ATCC 15305), *S. xylosus* (ATCC 29979), *S. hyicus* (ATCC 11249), *S. hominis* (ATCC 27844), *S. warneri* (ATCC 10209), *S. simulans* (ATCC 27851), and *S. haemolyticus* (ATCC 29970).

### 4.2. Detection of Enterotoxin and tst-1 Genes

PCR for the detection of the enterotoxin and *tst-*1 genes was performed according to the parameters described by Johnson *et al*. [28] and Cunha *et al*. [5].

The following toxigenic *S. aureus* reference strains were used as positive control: ATCC 13565 (*sea*; *ser*), ATCC 14458 (*seb*), ATCC 19095 (*sec*), ATCC 23235 (*sed*), ATCC 27664 (*see*), *S. aureus* Food Research Institute-FRI 361 (*sei*), and ATCC 51650 (*tst*). *Staphylococcus xylosus* ATCC 29971 was used as the negative control. The primer sequences are shown in Table 3.

### 4.3. Analysis by Pulsed-Field Gel Electrophoresis

The *Staphylococcus* spp. isolates that were positive for the enterotoxins by PCR were submitted to clonal profile analysis by pulsed-field gel electrophoresis (PFGE) according to the modified protocol of McDougal *et al*. [33].

The BioNumerics software (Version 7.0, Applied Maths, Sint-Martens-Latem, Belgium, 2015) was used for similarity analysis. Dice correlation coefficients were calculated and a dendrogram was generated using the UPGMA method (unweighted pair group method using arithmetic averages). Band position tolerance and optimization were set at 1.25% and 1%, respectively. A similarity coefficient of 80% was chosen for the definition of clusters.

### 4.4. Spatial Distribution of Staphylococcal Enterotoxin Genes in Different Regions of Brazil

The addresses of all isolates were geocoded and added to the BioNumerics program (Version 7.0, Applied Maths, Sint-Martens-Latem, Belgium, 2015). Only strains carrying some staphylococcal enterotoxin genes or toxic shock syndrome were adopted as inclusion criteria on the map.

## 5. Conclusions

The enterotoxin A gene exhibited the highest prevalence in the present study, suggesting its easy dissemination among *Staphylococcus* spp. strains, since it was widespread in the georeferencing study. The recently described enterotoxin R gene is already found in isolates from some of the regions in Brazil and coexists with previously described enterotoxins. This fact poses a risk for the dissemination of this new type of enterotoxin among cattle herds. A limitation of the present study was the fact that we did not perform gene expression analysis since the simple presence of enterotoxin genes in *Staphylococcus* spp. isolates does not imply the occurrence of food poisoning. However, the present results contribute to the understanding of the enterotoxigenic profile of isolates from cows with bovine subclinical mastitis in different Brazilian herds, especially when considering that they are one of the most common microorganisms involved in intramammary infections in cattle and that milk is an excellent growth medium for *Staphylococcus* spp*.* The study also identified important *S. aureus* and *S. epidermidis* clusters in different regions and the presence of enterotoxin genes in lineages isolated from animals that belong to herds located geographically close to each other.

## Figures and Tables

**Table 1:** Number of strains evaluated (*n*) and detection of enterotoxin genes in *Staphylococcus* species isolated from cows with bovine subclinical mastitis in different Brazilian states.

Species	(*n*)	*sea*	*seb*	*sec*	*sed*	*see*	*seg*	*seh*	*sei*	*sej*	*ser*	*ses*	*set*	*tst-*1
*S. aureus*	(82)	15	9	6	0	4	1	21	9	0	3	0	0	0
*S. chromogenes*	(27)	5	0	5	0	1	0	10	1	0	0	0	0	0
*S. epidermidis*	(26)	3	2	8	1	4	1	6	0	0	0	0	0	0
*S. saprophyticus*	(17)	5	1	1	0	2	0	4	0	0	0	0	0	0
*S. haemolyticus*	(6)	1	1	2	0	1	0	0	0	0	0	0	0	0
*S. simulans*	(6)	1	0	1	0	1	0	1	0	0	0	0	0	0
*S. warneri*	(6)	0	0	2	0	1	0	2	1	0	0	0	0	0
*S. hyicus*	(5)	2	1	1	0	1	1	1	1	0	0	0	0	0
*S. hominis*	(4)	0	0	0	0	0	0	1	0	0	0	0	0	0
*S. xylosus*	(2)	1	0	1	0	0	0	0	0	0	0	0	0	0
Total	181	33	14	27	1	15	3	46	12	0	3	0	0	0

*sea*: staphylococcal enterotoxin A gene; *seb*: staphylococcal enterotoxin B gene; *sec*: staphylococcal enterotoxin C gene; *sed*: staphylococcal enterotoxin D gene; *see*: staphylococcal enterotoxin E gene; *seg*: staphylococcal enterotoxin G gene; *seh*: staphylococcal enterotoxin H gene; *sei*: staphylococcal enterotoxin I gene; *sej*: staphylococcal enterotoxin toxin J gene; *ser*: staphylococcal enterotoxin R gene; *ses*: staphylococcal enterotoxin S gene; *ser*: staphylococcal enterotoxin R gene; *set*: staphylococcal enterotoxin T gene; *tst*: toxic shock syndrome toxin 1 gene.

**Table 2:** Detection of enterotoxin genes in *Staphylococcus aureus* and *Staphylococcus epidermidis* clusters according to the region studied (PR: Paraná; SP: São Paulo; SC: Santa Catarina; RS: Rio Grande do Sul).

Species	Cluster	No. of Isolates	Enterotoxin Genes	Origin of Isolates
*S. aureus*	A	7	*sea* (7); *seb* (7); *sec* (1); *seh* (5); *sei* (2)	PR; SP
	B	4	*sea* (2); *seb* (1); *sec* (1); *see* (1); *seg* (1); *seh* (1); *sei* (1)	SC; RS
	C	3	*sea* (1); *see* (1); *seh* (1); *sei* (1)	SC
*S. epidermidis*	A	3	*sea* (2); *seb* (1); *sec* (2); *see* (1); *seh* (2)	SC
	B	3	*seb* (1); *sec* (1); *see* (2)	PR

**Table 3:** Sequence of the primers used and amplicon size.

Name	Product	5′ to 3′ Nucleotide Sequence	Reference	Amplicon Size (bp)
*sea*1	Enterotoxin A	TTGGAAACGGTTAAAACGAA	[28]	120
*sea*2		GAACCTTCCCATCAAAAACA		
*seb*1	Enterotoxin B	TCGCATCAAACTGACAAACG	[28]	478
*seb*2		GCAGGTACTCTATAAGTGCC		
*sec*1	Enterotoxin C	GACATAAAAGCTAGGAATTT	[28]	257
*sec*2		AAATCGGATTAACATTATCC		
*sed*1	Enterotoxin D	CTAGTTTGGTAATATCTCCT	[28]	317
*sed*2		TAATGCTATATCTTATAGGG		
*see*1	Enterotoxin E	CAAAGAAATGCTTTAAGCAATCTTAGGCCAC	[29]	170
*see*2		CTTACCGCCAAAGCTG		
*seg*1	Enterotoxin G	AATTATGTGAATGCTCAACCCGATC	[29]	642
*seg*2		AAACTTATATGGAACAAAAGGTACTAGTTC		
*seh*1	Enterotoxin H	CAATCACATCATATGCGAAAGCAG	[30]	375
*seh*2		CATCTACCCAAACATTAGCACC		
*sei*1	Enterotoxin I	CTCAAGGTGATATTGGTGTAGG	[29]	576
*sei*2		AAAAAACTTACAGGCAGTCCATCTC		
*selj*1	Enterotoxin J	CATCAGAACTGTTGTTCCGCTAG	[31]	146
*selj*2		CTGAATTTTACCATCAAAGGTAC		
*ser*1	Enterotoxin R	AGATGTGTTTGGAATACCCTAT	[32]	123
*ser*2		CTATCAGCTGTGGAGTGCAT		
*ses*1	Enterotoxin S	TTCAGAAATAGCCAATCATTTCAA	[8]	195
*ses*2		CCTTTTTGTTGAGAGCCGTC		
*set*1	Enterotoxin T	GGTGATTATGTAGATGCTTGGG	[8]	170
*set*2		TCGGGTGTTACTTCTGTTTGC		
*tst*1	Toxic shock syndrome toxin	ATGGCAGCATCAGCTTGATA	[28]	350
*tst*2		TTTCCAATAACCACCCGTTT		

## References

[1] Radostitis O.M., Gay C.C., Hinchcliff K.W., Constable P.D. (2007). Veterinary medicine. A Textbook of the Disease of Cattle, Horses, Sheep, Pigs and Goats.

[2] Rall V.L., Miranda E.S., Castilho I.G., Camargo C.H., Langoni H., Guimarães F.F., Júnior J.P.A., Júnior A.F. (2014). Diversity of *Staphylococcus* species and prevalence of enterotoxin genes isolated from milk of healthy cows and cows with subclinical mastitis. J. Dairy Sci..

[3] Karahan M.N., Açik B. (2009). Çetinkaya investigation of toxin genes by polymerase chain reaction in *Staphylococcus aureus* strains isolated from bovine mastitis in Turkey. Foodborne Pathog. Dis..

[4] Calsolari R.A.O., Pereira V.C.P., Júnior J.P.A., Cunha M.L.R.S. (2011). Determination of toxigenic capacity by RT-PCR in coagulase-negative staphylococci and *Staphylococcus aureus* isolated from newborns in Brazil. Microbiol. Immunol..

[5] Cunha M.L.R.S., Peresi E., Calsolari R.A.O., Júnior J.P.A. (2006). Detection of enterotoxins genes on coagulase-negative staphylococci isolated from foods. Braz. J. Microbiol..

[6] Omoe K., Imanishi K., Hu D.L., Kato H., Takahashi-Omoe H., Nakane A., Uchiyama T., Shinagawa K. (2004). Biological properties of staphylococcal enterotoxin-like toxin type R. Infect. Immun..

[7] List of Prokaryotic Names with Standing in Nomenclature—Genus *Staphylococcus*. http://www.bacterio.net/.

[8] Ono H.K., Omoe K., Imanishi K., Iwakabe Y., Hu D.L., Kato H., Saito N., Nakane A., Uchiyama T., Shinagawa K. (2008). Identification and characterization of two novel staphylococcal enterotoxins, types S and T. Infect. Immun..

[9] Borst D.W., Betley M.J. (1994). Phage-associated differences in staphylococcal enterotoxin A gene (*sea*) expression correlate with sea allele class. Infect. Immun..

[10] Evenson M.L., Hinds M.W., Bernstein R.S., Bergdoll M.S. (1988). Estimation of human dose of staphylococcal enterotoxin A from a large outbreak of staphylococcal food poisoning involving chocolate milk. Int. J. Food Microbiol..

[11] Balaban N., Rasooly A. (2000). Staphylococcal enterotoxins. Int. J. Food Microbiol..

[12] Tremaine M.T., Brockman D.K., Betley M.J. (1993). Staphylococcal enterotoxin A gene (*sea*) expression is not affected by the accessory gene regulator (*agr*). Infect. Immun..

[13] Marr J.C., Lyon J.D., Roberson J.R., Lupher M., Davis W.C., Bohach G.A. (1993). Characterization of novel type C staphylococcal enterotoxins: Biological and evolutionary implications. Infect. Immun..

[14] Bayles K.W., Iandolo J.J. (1989). Genetic and molecular analyses of the gene encoding staphylococcal enterotoxin D. J. Bacteriol..

[15] Kokan N.P., Bergdoll M.S. (1987). Detection of low-enterotoxin-producing *Staphylococcus aureus* strains. Appl. Environ. Microbiol..

[16] Veras J.F., Simeão L.C., Lawrence C.T., Jefrey W.S., Christiano C., Santos D.A., Cerqueira M.M.O.P., Cantini A., Nicoli J.R., Jett M. (2008). A study of the enterotoxigenicity of coagulase-negative and coagulase-positive staphylococcal isolates from food poisoning outbreaks in Minas Gerais, Brazil. Int. J. Infect. Dis..

[17] Srinivasan V., Sawant A.A., Gillespie B.E., Headrick S.J., Ceasaris L., Oliver S.P. (2006). Prevalence of enterotoxin and toxic shock syndrome toxin genes in *Staphylococcus aureus* isolated from milk of cows with mastitis. Foodborne Pathog. Dis..

[18] Van den Bussche R.A., Lyon J.D., Bohach G.A. (1993). Molecular evolution of the staphylococcal and streptococcal pyrogenic toxin gene family. Mol. Phylogenet..

[19] Lawrynowicz-Paciorek M., Kochman M., Grochowska A., Windyga B. (2007). The distribution of enterotoxin and enterotoxin-like genes in *Staphylococcus aureus* strains isolated from nasal carriers and food samples. Int. J. Food Microbiol..

[20] Akineden Ö., Annemüller C., Hassan A.A., Lämmler C., Wolter W., Zschöck M. (2001). Toxin genes and other characteristics of *Staphylococcus aureus* isolates from milk of cows with mastitis. Clin. Diagn. Lab. Immun..

[21] Buzzola F.R., Quelle L., Gomez M.I., Catalano M., Steele-Moore L., Berg D., Gentilini E., Denamiel G., Sordelli D.O. (2001). Genotypic analysis of *Staphylococcus aureus* from milk of dairy cows with mastitis in Argentina. Epidemiol. Infect..

[22] Almeida L.M.D., Mamizuka E.M., Cunha M.L.R.S.C., Zafalon L.F. (2009). Fatores de Virulência e Genes Regulatórios agr de Staphylococcus aureus e Outras Espécies Coagulase Positivas Isoladas de Mastites Bovina e Ovina.

[23] Koneman E.W., Allen S.D., Janda W.M., Schreckenberger P.C., Winn W.C. (1997). Color Atlas and Textbook of Diagnostic Microbiology.

[24] Cunha M.L.R.S., Sinzato Y.K., Silveira L.V.A. (2004). Comparision of methods for identification of Coagulase-negative Staphylococci. Mem. Inst. Oswaldo Cruz.

[25] Barry T., Colleran G., Glennon M., Dunican L.K., Gannon F. (1991). The 16S/23S ribosomal spacer region as a target for DNA probes to identify eubacteria. PCR Methods Appl..

[26] Couto I., Pereira S., Miragaia M., Sanches I.S., Lencastre H. (2001). Identification of clinical staphylococcal isolates from humans by Internal Transcribed Spacer PCR. J. Clin. Microbiol..

[27] Straub J.A., HerteL C., Hammes W.P. (1999). A 23S rDNA-targeted polymerase chain reaction-based system for detection of *Staphylococcus aureus* in meat starter cultures and dairy products. J. Food Prot..

[28] Johnson W.M., Tyler S.D., Ewan E.P., Ashton F.E., Pollard D.R., Rozee K.R. (1991). Detection of genes for enterotoxins, exfoliative toxins, and toxic shock syndrome toxin 1 in *Staphylococcus aureus* by the polymerase chain reaction. J. Clin. Microbiol..

[29] Jarraud S., Cozon G., Vandenesch F., Bes M., Etienne J., Lina G. (1999). Involvement of enterotoxins G and I in staphylococcal toxic shock syndrome and staphylococcal scarlet fever. J. Clin. Microbiol..

[30] Jarraud S., Mougel C., Thioulouse J., Lina G., Meugnier H., Forey F. (2002). Relationships between *Staphylococcus* genetic background, virulence factors, *agr* groups (alleles), and human disease. Infect. Imunn..

[31] Monday S.R., Bohach G.A. (1999). Use of multiplex PCR to detect classical and newly described pyrogenic toxin genes in staphylococcal isolates. J. Clin. Microbiol..

[32] Chiang Y.C., Liao W.W., Fan C.M., Pai W.Y., Chiou C.S., Tsen H.Y. (2008). PCR detection of staphylococcal enterotoxins (SEs) N, O, P, Q, R, U, and survey of SE types in *Staphylococcus aureus* isolates from food-poisoning cases in Taiwan. Int. J. Food Microbiol..

[33] McDougal L.K., Steward C.D., Killgore G.E., Chaitram J.M., McAllister S.K., Tenover F.C. (2003). Pulsed-field gel electrophoresis typing of oxacillin-resistant *Staphylococcus aureus* isolates from the United States: establishing a national database. J. Clin. Microbiol..

